# Foreign Correspondence

**Published:** 1855-09

**Authors:** 


### SELECTIONS.

*Foreign Correspondence of the New Hampshire Journal of Medicine.*

BERLIN, May 22, 1855.

MY DEAR SIR:—I promised the readers of the Journal, in my first letter, to give some accounts of the facilities which the city of Berlin presents as a medical centre. A harder task could scarcely be chosen, for I find after having been here now more than a month, that I have not as yet become acquainted with one half the advantages which are afforded to the student of our profession.

It has become common in America, as you know, to regard Paris as the great medical capital of Europe. This is undoubtedly true, but I am convinced that the large German cities are, in this respect, greatly underrated. They are, to be sure, further from us, and not so accessible from America. The German language is not so readily acquired as the French; and, more than all, our countrymen have, of late years, more and more fallen into the habit of going to Paris to complete a medical education. There is no question, however, but that Berlin, Vienna, and Prague will, in a very few years, become the resort of all who wish to pursue their studies, for a length of time, upon the continent.

The chief attractions of Berlin, in a medical point of view, are the public and private hospitals, and the lectures in the Royal University. The city itself, having a population of over four hundred thousand, affords abundant material to the student of every class of disease. The hospitals are immense, and among the best regulated in Europe, and the private and public clinics are unsurpased in the world A peculiar feature of this city is the great number of private institutions, devoted to special disease, to which the foreign student can obtain an easy access, if he desire it.

It so happened that the time of my arrival here was very fortunate. The summer's term, or semester of the University, had just opened, and the various courses of lertures had hardly commenced.

The method of study here, is in many respects widely different from the system adopted in America. There are none of those isolated medical schools, as with us, having at most seven or eight professors, and hardly able to subsist on account of the competition of other schools. The different professions are, on the contrary, here all represented in the universities; the lectures are given simultaneously, and degrees are conferred at the same time in all. The number of professors in the Medical Faculty of the university at Berlin is forty, and the students are at liberty to attend the lectures of such as they prefer. Oftentimes, there are four or five courses upon the same subject going on at once.

This method has great advantages. The schools have greater influence upon the profession of the country. The facilities, which in our country, are divided around among a score of little institutions, are all concentrated in one place. The museums are larger, and there is a natural emulation among the professors to excel, as they thus obtain a greater number of students at their lectures. The students may, at the same place, hear the same topics illustrated by several men, and thus acquire a much more comprehensive view of his studies, than where he is obliged, to gain the same advantage, to go hundreds of miles to hear a different corps of professors.

The great advantage, however, of the European system is, that all of the Professors are resident. Many have their private hospitals or clinics, and the student of a specialty may carry his investigation as far as he chooses, and find an unlimited means of illustration. At his examination for a medical degree, however, all of the Professors take a part, and the student has his real knowledge tested far more severely, than when graduating fees, or rivalry between schools, are the elements of success.

Yet Berlin is no place for the foreign student to begin his studies. One needs the stimulant, which he receives from a comparison of the unusal facilities which are offered in European institutions, and the higher scale of learning here, with the thorough, though not so extensive course of instruction in our ordinary medical schools at home.

I can give the readers of the Journal no better idea of the extent of the course of study in Berlin, than the fact, that there are over eighty separate courses of instruction given by the professors, during the present term. There are lectures upon General and Special Surgery, Anatomy, Pathology, Obstetrics, Physiology and Therapeutics. Besides these, there are courses upon all of those collateral branches which are now as necessary to a thorough medical education as any of those ordinarily taought in our schools. There are those upon Forensic Medicine, the History of Medicine, Microscopy, Botany, Toxicology, Comparative Anatomy and Pharmacy.

Special courses of instruction are also given upon Diseases of the Ear and Eye—and the profession of Berlin is at the head of the world in this department—upon Diseases of Women and Children, abdominal and cutaneous diseases, auscultation and percussion, and diseases of the brain.

Whenever it is possible to introduce practical instruction, this is done. Botanical excursions are made with students; they have an opportunity of performing all the operations of surgery upon the cadaver; lectures are given in the museums, in the dead houses attached to the hospital and in private laboratories. The lectures continue daily from six o'clock in the morning till eight at night, for eight months of the year. There are two terms annually, and the student, in pursuing a five or six years' course, can have an opportunity of gaining a practical knowledge of his profession, from the adjustment of a bandage to the diagnosis of a grave disease.

The two most prominent men in the Medical Faculty of Berlin are, probably, Langenbeck and Von Græfe. The former, the successor of Dieffenbach, as a surgeon, is second to no one in Europe, and his name is now quoted as the first surgical authority of Germany. The few American surgeons who have visited this city and made his acqaintance, will always remember the attentions with which he receives our countrymen, and the pleasure and instruction which his conversation and public clinics afford. In personal appearance, Prof. Langenbeck is altogether prepossessing. He is a slight built man, of about forty years, of a thoughtful countenance, and wearing the look of one who is constantly active but never exhausted. He speaks English perfectly, and often refers to the labors of English surgeons with evident admiration.

I was surprised to find him so well acquainted with the names and success of the distinguished men of his profession in America. While spending an evening lately at his beautiful residence in the Thier Garten, he took occasion to express himself in the warmest manner in regard to Drs. Pancoast, Mott, Carnochan, Mussey and Warren. He spoke of Mr Guthrie's first idea of chloroform, and the discovery of the application and advantage of ether in surgery, as entirely originating with us. He praised the success of our surgeons in many operations, in which those of Europe are quite unfortunate, and I remember his speaking, in particular, of the operations of his personal friend, Dr. Kimball of Lowell, in abdominal surgery, as being altogether unsurpassed upon the continent Dr. Langenbeck has had but seven cases of ovarian tumor in which he has performed the long Cæsarian section, and five of these died of secondary Peritonitis. In none of them, was there any thing to counter-indicate the operation. Dr. L. thinks that the influence of climate is of great weight in the success or failure of this class of cases. It is worth a remark, that Dr. Langenbeck's uncle was successful in extirpating one uterus, but this was taken out below, and not by the abdominal section. He regards the celebrated case of Dr. Kimball as unprecedented in surgery.

Prof. Langenbeck is himself a cautious surgeon. In cancerous affection of the mamma, particularly, he is never in a hurry to operate till the disease is quite developed, and in all malignant cases, he refrains from too early a use of the knife. I mention this fact as interesting, when the profession is so eager to learn the opinions of the best surgeons upon these points.

The treatment of surgical diseases by Langenbeck is often original. In hernia humoralis, for example, although compression and the Iodine Ungent are used by him. his favorite remedy is the frequent introduction of a bougie into the urethra. He has had one or two cases, apparently of carcionoma of the testis, prove themselves to be non-malignant, by being thus entirely cured. In the military hospitals here, where this disease not unfrequently occurs in patients who have suffered from tedious rides on horseback, I am told that strapping the testicle tightly with strips of plaster, prepared with the addition of some resolvent medicine, succeeds admirably. The plan is very nearly the same as recommended in Mr. Furgusson's system of surgery, when the adhesive plaster is combined with the camphorated mercurial plaster.

The surgical clinics of Berlin are full of interest, and Langenbeck's in particular. This is attended by hundreds of the students from all parts of Europe, who are attracted by the fame which he has acquired, and by his skill in operating. He is at the head of the Royal Opthalmic Institution here, and holds a clinic of two hours in length daily. I can give your readers no better idea of their character than by mentioning a few of the interesting cases. Each one was commented on for fifteen or twenty minutes by the Professor as it was presented before him.

1. Young lady ; woke up three days since, with loss of movement in the right shoulder and great pain. There is now crepitation. Diagnosis; a chronic inflammation which has been going on sometime, and which now manifiests itself. Treatment, by local counter irritation, tonics, and eventually passive motion. The result to be feared is, of course, anchylosis.

2. Hare lip in a child a few weeks old. Prof. Langenbeck adopts the principle which the London and Edinburgh surgeons are just now so zealously unholding, of operating in these cases as early as possible. He uses the common interupted suture almost invariably.

3. Osteo sarcoma of the scapula. Removed by an operation.

4. Young man, 26 years old, whose right testicle is still contained in the inguinal canal. The patient objects to being operated upon.

5. Child with two thumbs on each hand ; one removed at the desire of the mother.

6. Carcinoma in a woman of 63. From the left external labium, one tumor is extracted as large as a pear, and a smaller one from each groin.

7. Child 2 years old. Clubbed hands. The tendons of the flexors at fault are cut, and an apparatus ordered.

8. Hydrocele in a middle aged man. Injected with diluted chloroform.

Chloroform, by the way, is used altogether as an anæsthetic agent in Berlin, and with few bad results Langenbeck informs me, that although he has had several cases of deep asphyxia, he has had only one case of death, in his whole practice, from its use.

But I must draw this long letter to a conclusion. I will simply take leave of you and the readers of the Journal, in giving the details of a most singular case which has lately occurred to the distinguished man, of whom I have endeavored to convey a practical portrait. I have just been looking at the plates which represent it in the portfolio of Prof. L. The history of the case is simply this. The patient, a young lady, was suddenly affected by a suppression of her menstrual discharges, and soon after, several cruptive spots came out, first on one cheek and then on the other, and finally upon the nose. The eruption resembled lupus, but was evidently different. It destroyed the entire nasal organ. A new nose was formed from a flap taken from the forehead by the Taliacotian operation. The result was union by the first intention. *The menses reappeared at once after the operation*, and Dr. L. congratulated the lady upon this fact, as he thought that between the eruption and the suppression, he discovered a connection. But the menses again stopped; the eruption reappeared, and the new nose entirely sloughed off The question of interest in the case is, of course, the nature of the eruption. N. E. G.

